# The endovenous laser treatment for patients with varicose veins

**DOI:** 10.12669/pjms.321.8610

**Published:** 2016

**Authors:** Jian-Jun Liu, Long-Hua Fan, De-Chun Xu, Xu Li, Zhi-Hui Dong, Wei-Guo Fu

**Affiliations:** 1 Jian-Jun Liu, Department of Vascular Surgery, Zhongshan Hospital Qingpu Branch, Fudan University, Shanghai 201700, China; 2 Long-Hua Fan, Department of Vascular Surgery, Zhongshan Hospital Qingpu Branch, Fudan University, Shanghai 201700, China; 3 De-Chun Xu, Department of Vascular Surgery, Zhongshan Hospital Qingpu Branch, Fudan University, Shanghai 201700, China; 4 Xu Li, Department of Vascular Surgery, Zhongshan Hospital Qingpu Branch, Fudan University, Shanghai 201700, China; 5 Zhi-Hui Dong, Department of Vascular Surgery, Zhongshan Hospital Fudan University, Shanghai 200032, China; 6 Wei-Guo Fu, Department of Vascular Surgery, Zhongshan Hospital Fudan University, Shanghai 200032, China

**Keywords:** Endovenous laser treatment, Great saphenous veins, Varicose veins

## Abstract

**Objective:**

To evaluate the clinical effect of endovenous laser treatment (EVLT) for patients with varicose veins.

**Methods:**

Our series included 117 patients who underwent EVLT combined with high ligation and stripping since the introduction of the technique in our institution. All EVLT procedures were performed with local skin cooling to prevent skin burns, as well as stripping after exsanguinations to prevent thrombotic phlebitis.

**Results:**

A total of 146 limbs in 117 patients were ablated by EVLT. Bilateral EVLT was performed in 29 patients, with the remaining 88 patients undergoing unilateral EVLT. The mean age of the patients was 57 years ± 12 years (range: 21 years to 80 years), and 56 were male and 61 were female. Follow-up for all patients lasted three to six months. The most common complication was induration and swelling, which was observed in 64 patients, followed by paraesthesia in 27, and skin burns in 12.

**Conclusion:**

The treatment with endovenous laser treatment for patients with varicose veins is safe and effective.

## INTRODUCTION

Varicose veins are a common clinical problem in vascular surgery that may significantly affect quality of life, with involvement as high as 10% to 40% of the population in China. However, different treatment methods have different results.^1^ In 1997, Bergan first applied endovenous laser treatment (EVLT) for varicose veins, which has been applied in China since 2003. EVLT is safe and effective,^2^-^4^ especially in terms of faster recovery.^5^ However, the performance standards for this technique have not been released to date. Consequently, inappropriate EVLT procedures increase the incidence of postoperative complications or even undermine the apparent advantage of this technique in terms of fast patient recovery. Nevertheless, it has been extensively applied in basic hospitals. Since 2011, we have explored and improved the technique to reduce postoperative complications based on the considerable work experience of others.

## METHODS

### Patients

The study included 117 patients treated for varicose veins with saphenous reflux, which consisted of 61 females and 56 males, with an age range of 21 years to 80 years (mean 57 years ± 12 years). The data of the patients who received EVLT from Jan. 2011 to Dec. 2013 in our center were analyzed retrospectively. Unilateral EVLT was performed in 88 patients (left 46 and right 42), with the remaining 29 undergoing bilateral EVLT. Our patients endured symptoms for two year to 35 years before seeking surgical treatment. The patients were classified as ‘clinical, aetiological, anatomical, and pathologic’ (CEAP), including 47 C3 patients (with varicose veins or swelling), 62 C4 patients (with pigmentation), 16 C5 patients (with active ulcer), and 12 C6 patients (with previous bleeding episodes). This study was conducted in accordance with the declaration of Helsinki and approval from the Ethics Committee of Zhongshan Hospital of Fudan University. Written informed consent was obtained from all participants.

The indications for treatment were based on patient preference. The most common indication for EVLT was large bulging varicosities and accompanying symptoms such as lower extremity swelling, eczema, pigmentation, stasis dermatitis, and ulceration. All patients underwent duplex scanning to document the patency of the deep veins.

### Technique

The patients received spinal anaesthesia (90 patients) or general anaesthesia (27 patients). We used an 810 nm semiconductor laser (Dornier MedTech, Italy) with 600 µm-diameter optical fibre^6^ (however, other authors think that EVLT using a 1,320 nm-laser achieves better clinical outcomes, as well as lower recurrence and recanalisation rates than EVLT using an 810 nm laser.).^7^ An 18 gauge needle, 0.035 in ultrasmooth guide wire, 4/5 F catheter was used.

The technique involves making a 2 cm incision near the inguinal ligament, and then exposing, ligating, and then cutting the great saphenous vein (GSV) and its tributaries 0.5 cm distal to the saphenofemoral junction. Venous access was obtained by puncture with an 18 G needle near the ankle or via direct cannulation of the distal end of the GSV. After entering the vein, a hydrophilic guide wire was inserted, followed by a catheter, and the guide wire was then replaced with a laser fibre. The pulse mode was set at 16 W at the thigh, 14 W at the knee and 12 W at the calf with a pullback rate of 3 mm to 5 mm pers. The mean energy was 6515 J (ranging from 1000 J to 11330 J), and increasing energy delivery had no significant effect on EVLT morbidity and complications for superficial venous insufficiency.^2^

### Postoperative management and follow-up

An elastic bandage was applied for three days to five days immediately after the procedure, followed by graduated compression stockings for one month to three months. Patients were advised to ambulate as early as possible and the sutures were removed at seven days to 10 days postoperatively. Further assessments were scheduled after one week, one month, three months, and then 6 months. Follow-up included any symptoms, residual and recurrent varicose veins determined by physical examination, as well as the closure and recanalisation of the GSV using ultrasound. Data were collected in the outpatient department. The follow-up rate was 73% (86/113) and the mean follow-up for all patients was five months.

## RESULTS

### General information

During the study period, we found that complaints were most apparent within the first four months after operation. However, the accumulated surgical experience and improved methods reduced the number of complications. The higher the CEAP classification was, the higher the incidence of complications was. Complications are shown in Table-I, some patients had more than one complication.

**Table-I:** Complicaitons after EVLT.

Complications	Limbs	%
Skin burns	12	10%
Induration	39	33%
Swelling	25	21%
Paresthesia	27	23%
DVT or PE	0	0%
Recanalisation	0	0%

### Follow-up

The follow-up of all patients lasted three months to six months. The most common complication was induration and swelling, which developed in 64 patients, followed by paraesthesia in 27, and skin burns in 12. The symptoms gradually subsided and disappeared within 2 months to three months. No deep vein thrombosis, pulmonary embolism, or recanalisation was detected by duplex scanning. Active ulcers healed within about two weeks after surgery and eczema gradually disappeared in one month. Doppler ultrasonography was performed for some patients at two weeks and at four weeks after surgery. The results showed great saphenous vein atresia without blood flow signals, thrombosis, and enhanced echogenic dots and bands in the lumen of the blood vessel, as well as a noticeably thickened, coarse blood vessel wall with damaged integrity. At six months, ultrasonography was again performed for some patients. No recanalisation was observed.

## DISCUSSION

EVLT has been widely used for varicose veins. Although different methods have different results, EVLT is at least as effective as surgery in treating varicosity of the GSV.^8^ The primary failure and recurrence in EVLT was not significantly different from that of surgery.^9^ EVLT is safe, and although more energy is used, this has not translated into higher complication rates.^3^,^10^ Based on the experience of others, we explored and improved the treatment.

Venous hypertension of the lower extremity from congenital incompetent valves, weak vein wall, and/or long standing appears to be the underlying pathophysiology for varicose veins. The classic treatment for varicose veins is high ligation with GSV stripping at the saphenofemoral junction (SFJ) with or without phlebectomy. This includes cutting and ligating the GSV and its tributaries, stripping the trunk of the GSV, and removing other local varicose veins.^5^ The main purpose of a variety of surgical methods for treating varicose veins is to eliminate varicose veins, with endovenous ablation effectively reducing symptoms of superficial venous insufficiency.^11^ Incomplete removal of the great saphenous vein is the most common cause recurrent varicose veins, hence, there is a need to relieve venous hypertension and prevent recurrence.^12^ EVLT combined with high ligation is based on the principles of the operation.^13^,^14^ High ligation is a valid means to discharge hypertension, where the optical fibre directly enters the GSV cavity, and then damage the endothelial and intimal layers, as well as promote blood vessel closure and fibrosis. It has two functions: it prevents the laser head from moving into and damaging deep veins and it prevents thrombus migration from superficial veins into deep veins and subsequent potential pulmonary embolism. Recanalisation has been reported after simple EVLT,^13^ although recanalisation has not been found by B-ultrasound in our centre. However, continued clinical and duplex follow-up is needed to assess its long-term efficacy.^15^ Saphenofemoral recurrences after simple EVLT require further^3^ investigations. High ligation and cutting GSV tributaries, as well as tangential repair of the femoral vein prevents the formation of vortex at SFJ. A clear fossa ovalis can also eliminate the recurrences that result from anatomical variations such as double GSVs. High ligation also eliminates hypertension from incompetent saphenofemoral valves, femoral vein and its tributaries. The rate of varicose vein recurrence in the EVLT without SFL group is similar to that in the EVLT with SFL group. Less neo-vascularisation occurred in the EVLT without SFL group, but more incompetent tributaries and early recanalisation at 5-year follow-up occurred in the EVLT without SFL group than in the EVLT with SFL group.^16^

EVLT treatment of varicose vein uses lasers that generate high-energy. The produced heat is absorbed by haemoglobin and the surrounding tissue, which then results in the formation of steam bubbles. The bubbles cause blood to boil and induce thermal injuries to the venous endothelium and intima. The venous lumen then undergoes thrombosis, organization, fibrosis, and finally occlusion. Histologic studies show that EVLT damages the endothelial and intimal layers, the internal elastic lamina, and the media, to some degree.

The GSV is deep in the thigh, and skin burning is rare. Unlike those in the thigh, the varicose veins in the calf are relatively superficial, and if the steam bubbles lose control in the vein wall, they directly damage the skin and cause skin burns. Injecting protective solution or using a pump prevents skin burns, but the operation is relatively complicated.

Reducing energy accumulation in the calf prevents skin burns. Our method is spraying saline onto the skin exposed to the laser. This approach significantly reduces the skin temperature, and it is simple and effective. The fibre moving speed can also be increased, or the mode of the laser can be changed from continuous emission to intermittent emission to reduce the energy and reduce skin burns.

More energy is required to destroy vein wall for EVLT if a large number of tortuous veins are in the calf; however, more energy is available to injure the surrounding tissue. Hence, thrombosis, swelling and pain, as well as phlebitis, occurs postoperatively, and patients need more time to recover, which ultimately remove the advantages of minimally invasive procedures. Moreover, venous occlusion was less likely if EVLT was used alone. Consequently, we combined EVLT with stripping to treat serious varicose veins.

We incorporated a tourniquet into EVLT based on classic surgery experience. The affected limb was raised one to two minutes after EVLT, a tourniquet was tied, and the varicose vein was stripped. The tourniquet should not be applied on the calf because it may cause thrombus migration into the deep venous system. Finally, the tourniquet reduces bleeding, facilitating the stripping of varicose veins.

In summary, EVLT is an effective treatment for varicose veins, with possible complications,^17^ such as burns, recurrence, phlebitis, pain, and others, due to incorrect operation. Our exploration and improvement of the technique effectively reduced the occurrence of complications while maintaining its efficacy.
